# Supplementary figures and images for: Lipoprotein concentrations over time in the intensive care unit COVID-19 patients: Results from the ApoCOVID study
Source: PLoS One. 2020 Sep 24;15(9):e0239573. doi: 10.1371/journal.pone.0239573 (PMC7514065; doi:10.1371/journal.pone.0239573)

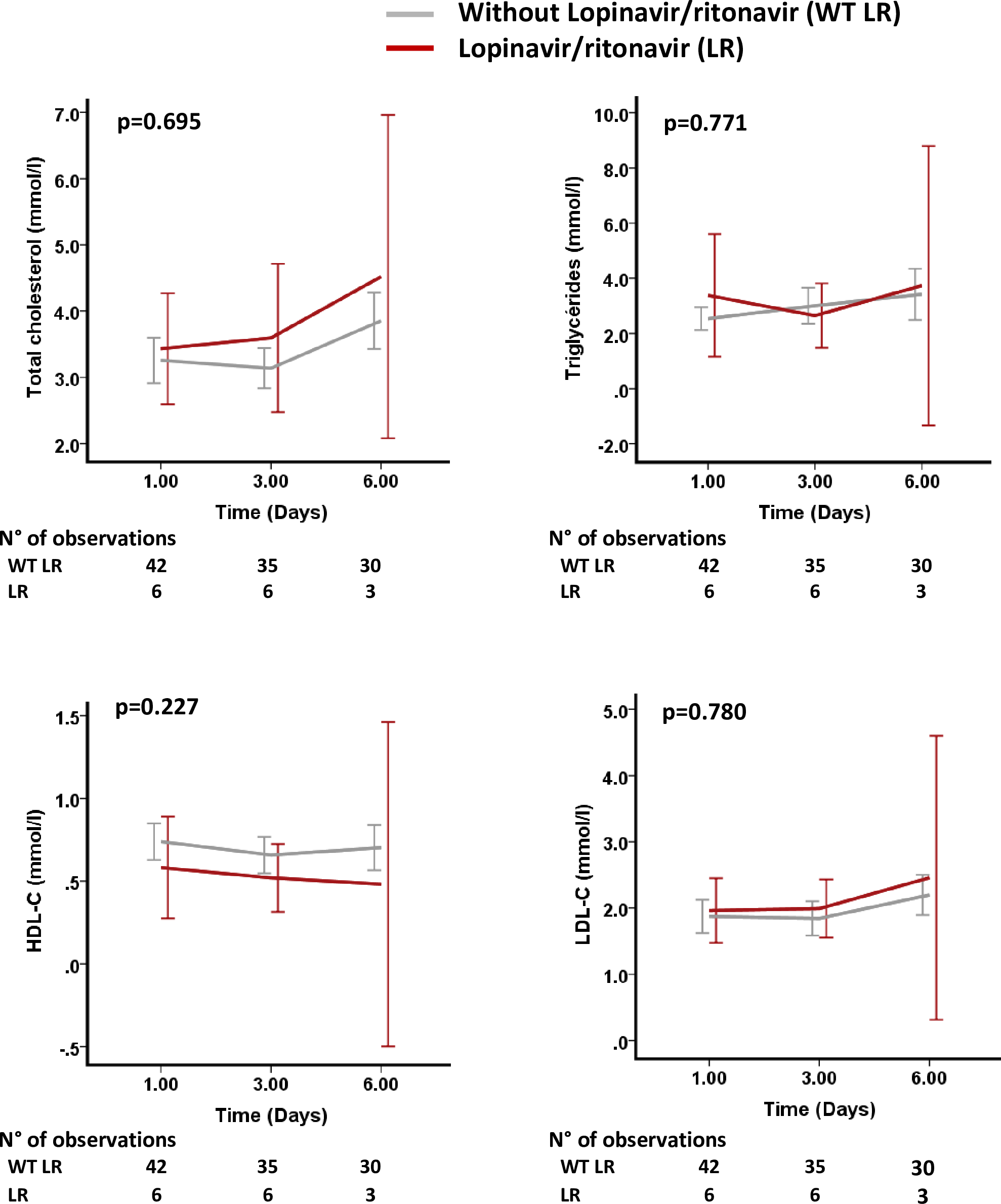

Supplement: S1 Fig — (TIF) [file pone.0239573.s001.tif]

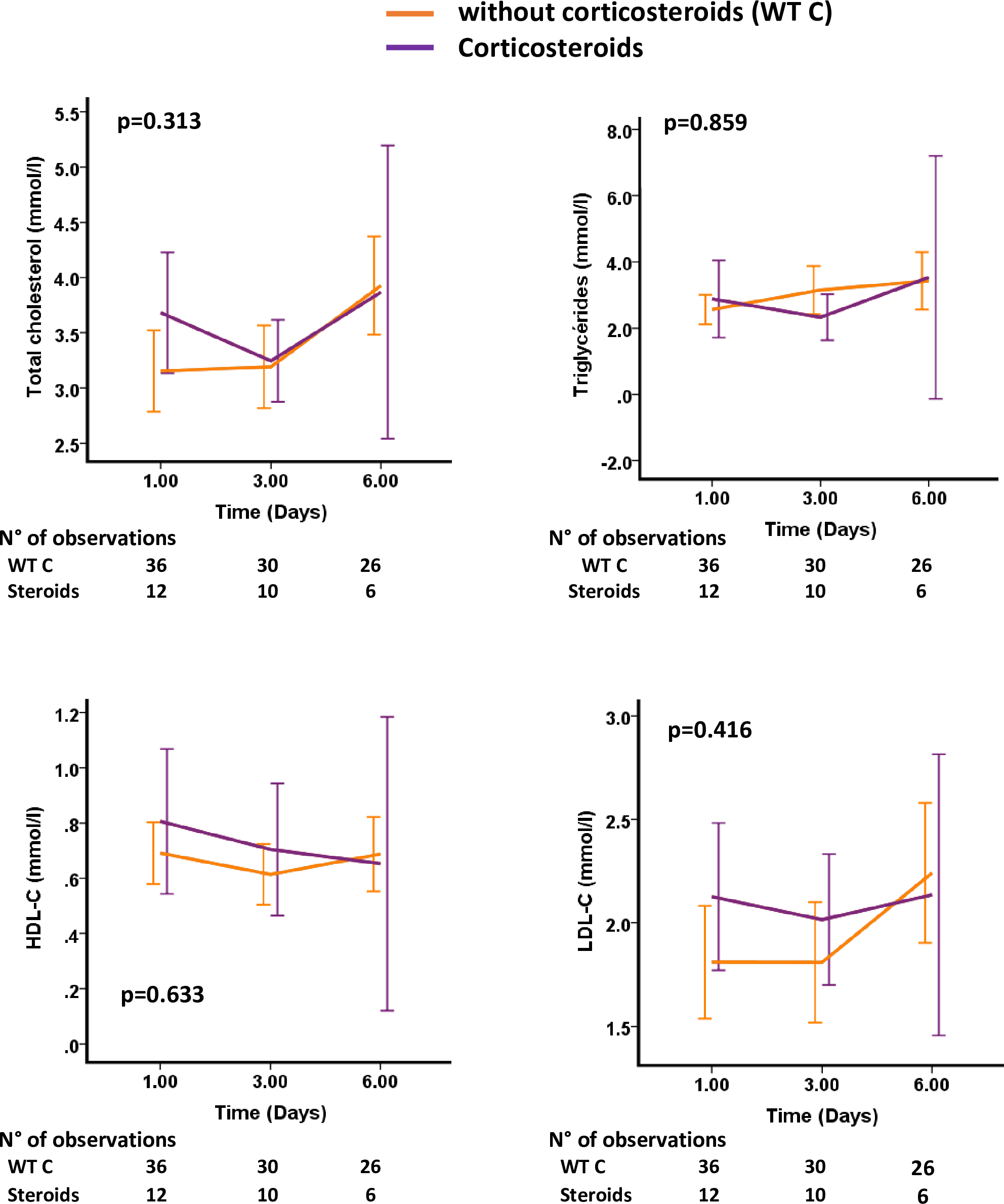

Supplement: S2 Fig — (TIF) [file pone.0239573.s002.tif]

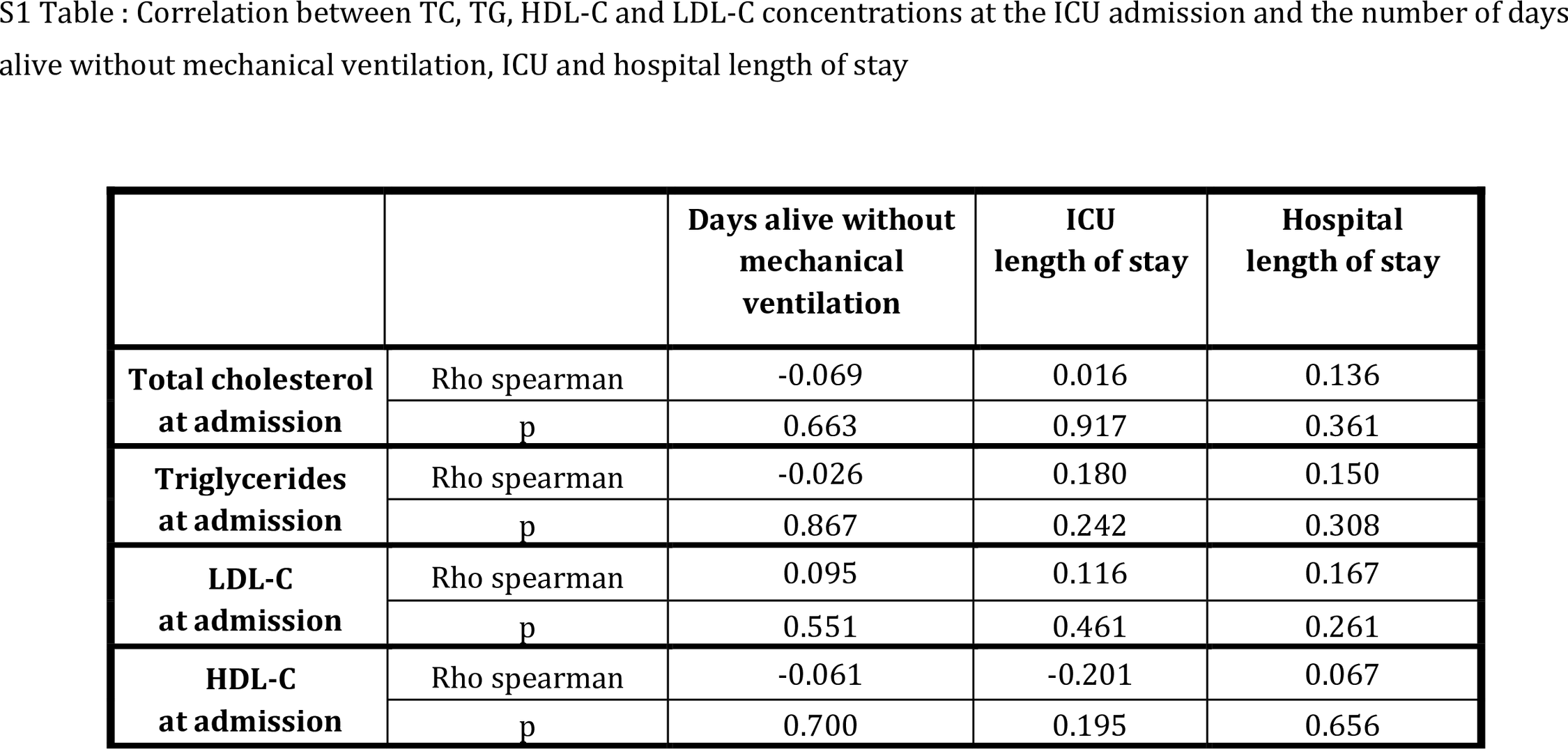

Supplement: S1 Table — (TIF) [file pone.0239573.s003.tif]
